# Preparation of Nano-Mg(OH)_2_ and Its Flame Retardant and Antibacterial Modification on Polyethylene Terephthalate Fabrics

**DOI:** 10.3390/polym15010007

**Published:** 2022-12-20

**Authors:** Ying Wang, Yanjing Liu, Xiyue Li, Yuezhou Liu, Fuming Wang, Yaping Huang, Lihua Lv, Ying Chu, Yongfang Qian

**Affiliations:** 1 School of Textile and Material Engineering, Dalian Polytechnic University, Dalian 116034, China; 2 China National Textile and Apparel Council Key Laboratory of Flame Retardancy Finishing of Textile Materials, Soochow University, Suzhou 215123, China

**Keywords:** nano-Mg(OH)_2_, flame retardant, antibacterial, PET fabrics

## Abstract

The multifunctional polyethylene terephthalate (PET) fabrics were successfully prepared through a dip-coating technology to endow the flame retardant and antibacterial properties of PET fabrics, which are extensively used in many fields. The flame retardant and antibacterial agent was synthesized by a double drop-reverse precipitation method and surface-modified by the mixtures of titanate coupling agents and stearic acid to result in a good compatibility of the hydrophilic nano-Mg(OH)_2_ and the hydrophobic PET fabrics. The results indicated that the suitable synthesis conditions of nano-Mg(OH)_2_ are: Mg^2+^ concentration 1.5 mg/mL, reaction temperature 50 °C and reaction time 50 min, and the optimal modification conditions of nano-Mg(OH)_2_ are: modifier ratio 5/5, modification temperature 70 °C and modification time 40 min. The flame retardant test and the antibacterial test showed that the multifunctional PET fabrics had excellent flame retardant and antibacterial properties.

## 1. Introduction

The polyethylene terephthalate (PET) fabrics, as the most common synthetic fibers, are widely used in a lot of fields due to their excellent stability, friction resistance and mechanical property [1,2,3,4]. However, the PET fabrics are flammable materials due to the limiting oxygen index (LOI) of 20–22%, and the heavy casualties and the huge economic losses caused by the PET burning fires occur frequently [5,6]. Additionally, the PET fabrics have no inherent resistance against bacteria, and the uncontrolled bacteria on the PET fabrics can seriously lead to abominable effects, such as disease, discoloration and malodor [7,8,9]. Thus, the high flammability and the no antibacterial group of the PET fabrics restrict the application scope, especially those where flame retardant and antibacterial properties are required, so that the treatment including flame retardant and antibacterial properties to the PET fabrics is necessary. 

Nano magnesium hydroxide (nano-Mg(OH)_2_) is an inorganic material with nanometer size, which has advantages such as large specific surface area, non-toxic performance, chemical stability and thermal stability [10,11,12]. As halogen-free and phosphorus-free flame retardants, nano-Mg(OH)_2_ has high decomposition temperature and soft texture, and especially has smoke suppression, which has good development prospects [13,14]. Nano-Mg(OH)_2_ can absorb a lot of heat during decomposition at a high temperature, thereby reducing the temperature of the combustion product and slowing down the combustion reaction [15]. The decomposition product MgO is also a high temperature-resistant substance, which could cover on the surface of PET fabrics to significantly improve the air isolation efficiency and further prevent combustion [16]. After decomposition, a large amount of water vapor is generated, which consumes part of the heat and dilutes combustible gases such as CO to a certain extent. Furthermore, nano-Mg(OH)_2_ can also absorb smoke and plays a role in eliminating smoke [17]. In addition, the nano-Mg(OH)_2_ has been found to have a broad-spectrum antibacterial property, which is representative of inorganic antibacterial material due to its excellence in stability and persistent antibacterial property, and the nano-Mg(OH)_2_ has great antibacterial property for loading on PET fabrics [18,19,20]. It has been reported that nano-Mg(OH)_2_ has antibacterial properties even in the dark, which implies that nano-Mg(OH)_2_ can be effective without light irradiation [21]. The antibacterial mechanism of nano-Mg(OH)_2_ is commonly attributed to the production of reactive oxygen species (ROS) on nano-Mg(OH)_2_ surface, which can cause bacterial lipid peroxidation and death [22,23,24]. Thus, the nano-Mg(OH)_2_ as flame retardant and antibacterial agent is suitable for loading on PET fabrics.

Among the methods which are commonly used for nano-Mg(OH)_2_ synthesis are sol-gel technique, microwave/ultrasound-assisted technique, precipitation of a magnesium salt with an alkaline solution and solvothermal treatment [25,26,27]. The microstructure of the nano-Mg(OH)_2_, i.e., the particle size, shape and agglomeration are crucial in flame retardant and antibacterial applications. The double-dropping technique could improve the instantaneous supersaturation of the reactants in the reaction system, which is beneficial to the formation of the particles with a uniform particle size [28,29]. The conventional chemical precipitation method is to add an alkaline substance as a precipitant to the salt solutions, while the reverse precipitation method is to drop the magnesium salt solutions into the sodium hydroxide alkaline solutions for the reaction, and the pH value is always higher than the isoelectric point of the Mg(OH)_2_ in water [30,31]. During the precipitation process, the net charge on the surface of the Mg(OH)_2_ crystal nucleus is always negative, and the electrostatic repulsion between the negative charges will prevent the particles from agglomerating.

In this work, the nano-Mg(OH)_2_ as flame retardant and antibacterial agent is synthesized by double drop-reverse precipitation method and surface-modified by the mixtures of titanate coupling agents and stearic acid to result in a good compatibility of the hydrophilic nano-Mg(OH)_2_ and the hydrophobic PET fabrics. The modified nano-Mg(OH)_2_ is loaded on the PET fabrics through dip-coating technology. A variety of characterizations including X-ray diffraction (XRD), scanning electron microscopy (SEM), attenuated total reflection Fourier transform infrared spectroscopy (ATR-FTIR) and thermogravimetric analysis (TGA) were used to characterize the powders and the fabrics. Additionally, the flame retardant and antibacterial properties of the fabrics were tested in this research.

## 2. Materials and Methods

### 2.1. Materials

The magnesium chloride hexahydrate (MgCl_2_·6H_2_O) and the sodium hydroxide (NaOH) purchased from Shanghai McLean Biochemical Technology Co., Ltd. (Shanghai, China), were used to synthesize the nano-Mg(OH)_2_. The commercial nano-Mg(OH)_2_ (C-M) for comparison was purchased from Xuancheng Jingrui New Material Co., Ltd. (Xuancheng, China). The polyethylene glycol and the absolute ethanol were purchased from Shanghai McLean Biochemical Technology Co., Ltd. The titanate coupling agent and stearic acid were purchased from Nanjing Chuangshi Chemical Auxiliary Co., Ltd. (Nanjing, China), and Shanghai McLean Biochemical Technology Co., Ltd. (Shanghai, China). The PET fabrics were purchased from Wujiang Haixu Textile Co., Ltd. (Wujiang, China). The *Escherichia coli* (*E. coli*, ATCC 25922) purchased from Shanghai Luwei Technology Co., Ltd., (Shanghai, China). was used as model bacteria. The *E. coli* was grown aerobically in Luria-Bertani (LB) medium (tryptone 1%, NaCl 0.5%, yeast extract 0.5%, pH 7.2) at 37 °C, and the *E. coli* culture was maintained on LB agar plates (tryptone 1%, NaCl 0.5%, yeast extract 0.5%, agar 2%, pH = 7.2) at 37 °C. The reagents were all analytically pure without further treatment or purification.

### 2.2. Synthesis and Settling Rate Test of Nano-Mg(OH)_2_

The NaOH solutions (2 mol/L 50 mL) were added to a 100 mL volumetric flask, and then 1.0% polyethylene glycol (accounting for the mass ratio of MgCl_2_·6H_2_O) was added to a volumetric flask. After adding the rotor to the volumetric flask, 50 mL MgCl_2_·6H_2_O solutions were slowly added dropwise to the volumetric flask in a double dropwise manner. The Mg^2+^ concentration, reaction temperature and reaction time are shown in Table 1. The reacted suspensions were suction filtered, washed (twice with deionized water; twice with absolute ethanol) and dried at 60 °C for 3 h. Finally, the 15 groups of samples of synthetic nano-Mg(OH)_2_ were obtained, and the sample names are shown in Table 1. As shown from the label names are the M-M means (nano-Mg(OH)_2_ sample)-(Mg^2+^ concentration), the M-T means (nano-Mg(OH)_2_ sample)-(reaction temperature) and the M-t means (nano-Mg(OH)_2_ sample)-(reaction time).

The settling rate of the synthetic nano-Mg(OH)_2_ are expressed by the sedimentation volume. The synthetic samples (1 g M-M-0.5, M-M-1.0, M-M-1.5, M-M-2.0, M-M-3.0, M-T-20, M-T-30, M-T-40, M-T-50, M-T-60, M-t-20, M-t-30, M-t-40, M-t-50 and M-t-60) and the commercial nano-Mg(OH)_2_ (1 g C-M) were dissolved in 100 mL of deionized water, respectively. Then, the samples were ultrasonically shaken for 1 h. Finally, the dispersed powder suspensions were put into 100 mL measuring cylinder for observation, and the settling time and the settling volume were recorded for analysis.

### 2.3. Hydrophobic Modification and Activation Index Test of Nano-Mg(OH)_2_

The synthetic nano-Mg(OH)_2_ was modified using titanate coupling agent and stearic acid; 200 mL of ethanol and 10 g of nano-Mg(OH)_2_ were added into a three-necked flask for ultrasonic dispersion. The titanate coupling agent and stearic acid dissolved in ethanol were added dropwise to the nano-Mg(OH)_2_ suspensions. The suspensions were fully stirred (500 rpm), and then centrifuged, washed and dried (60 °C, 3 h) after constant temperature reaction for a certain period of time. The modifier ratio, reaction temperature and reaction time are shown in Table 2.

The 5 g (m) modified nano-Mg(OH)_2_ and 100 mL deionized water were added into a 200 mL beaker and stirred for 10 min, which was left to stand for 60 min horizontally. The remaining floating powders (m_1_) were taken out and dried at 100 °C. The activation index was H = m_1_/m.

### 2.4. Preparation of Functional PET Fabrics

The pure PET fabrics were pretreated by washing and rinsing, and then dried at 90 °C for 0.5 h. The 50, 100, 150, 200, 250 g/L flame retardants including commercial nano-Mg(OH)_2_ (CM), synthetic nano-Mg(OH)_2_ (M) and modified nano-Mg(OH)_2_ (GM) were loaded onto the PET fabrics by dip-coating method (30 ℃ for 30 min), and then the fabrics were dried at 90 °C for 3 h. The prepared fabrics were named as F-0, CM-50, CM-100, CM-150, CM-200 and CM-250; M-50, M-100, M-150, M-200 and M-250; GM-50, GM-100, GM-150, GM-200 and GM-250.

### 2.5. Characterizations

The purity and the average size of the crystallite powders were analyzed by X-ray diffraction (XRD, Rigaku D/max-2500/PC) using Cu Kα radiation (*λ* = 0.15418) at 25 mA and 40 kV, which was acquired from 5° to 90° with a step size of 0.05°/s and calculated by Scherrer equation shown as Equation (1) [32]:

(1) $$D=\frac{K\cdot \lambda }{B\cdot \cos\theta }$$

where *D* refers to the particle size (nm); *K* refers to the Scherrer constant (0.89); *λ* refers to the diffraction wavelength (0.15418Å); *B* refers to the half width of the diffraction peak; *θ* refers to the diffraction angle.

The micro morphology of the powders and fabrics was characterized by scanning electron microscopy (SEM, JSM 7500F) after coating with gold on the surface of the samples. The combined way among the powders and the fabrics was characterized by the attenuated total reflection Fourier transform infrared spectroscopy (ATR-FTIR, Spectrum 2) at a resolution of 4 cm^−1^ in a range of wave numbers from 400-4000 cm^−1^. Moreover, the thermal behavior was tested by thermogravimetric analysis (TGA, TGA 2), which was performed at a heating rate of 10 °C/min in the range of 40–800 °C under a nitrogen atmosphere with a flow rate of 20 mL/min.

### 2.6. Flame Retardant Performance Test of Fabrics

The vertical burning and limiting oxygen index (LOI) were tested according to GB/T5455–2014 and GB/T 5454–1997. The vertical burning test was tested using a vertical burning tester (YG815B). The PET fabric sample size was 300 × 89 mm, and the ignition time was 60 s (temperature: 10–30 °C; relative humidity: 30–80%). After ignition and reaching 60 s, the igniter was removed and turned off. Then, the timer was turned on to record the duration of continuous combustion. The fabric sample (150 × 50 mm) for testing LOI was placed in a glass covered with a mixture of nitrogen and oxygen flow. The upper end of the sample was ignited with an igniter, and then the minimum oxygen concentration to maintain the flaming combustion of the sample was recorded.

### 2.7. Antibacterial Test of Fabrics

The antibacterial rate (*I*) of the fabrics was tested by a shake-flask method according to the modified GB/T 20944.3–2008, GB/T 24346–2009 and AATCC 100–2004, which was calculated by Equation (2) [33]:

(2) $$I=\left[\frac{A-B}{A}\right]\times 100$$

where *I* refers to the antibacterial rate (%); *A* refers to the *E. coli* colonies number of control; *B* refers to the *E. coli* colonies in the number of the samples.

## 3. Results and Discussion

### 3.1. Characterization and Performance of Nano-Mg(OH)_2_

#### 3.1.1. XRD Result

**Figure 1:**
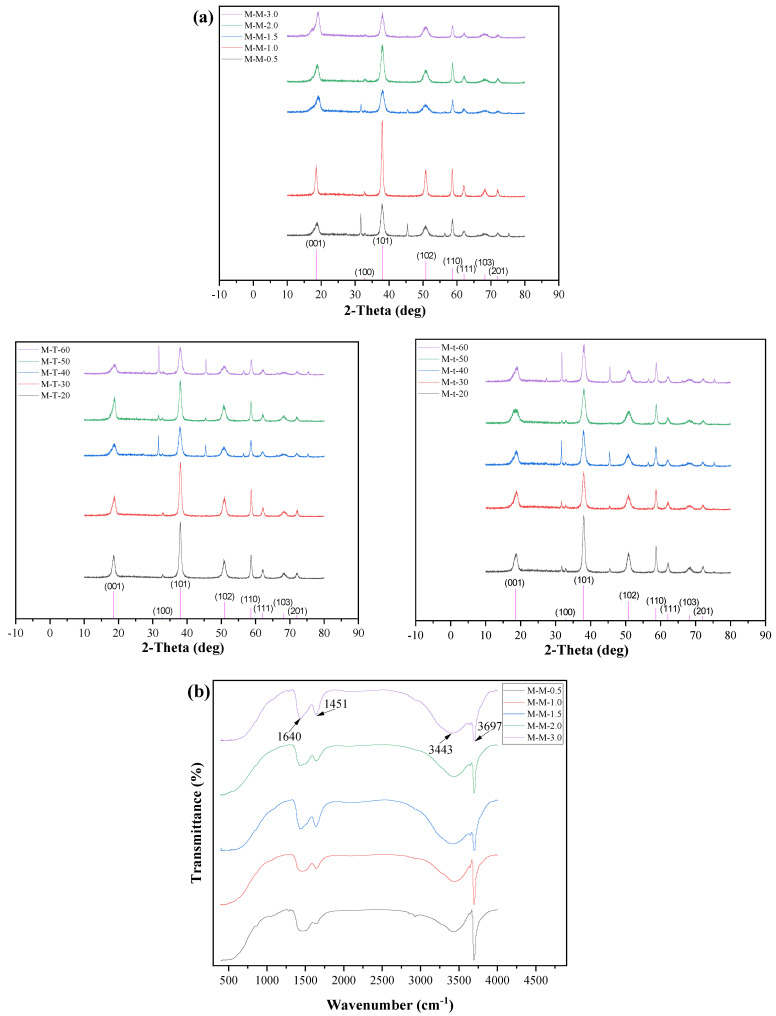

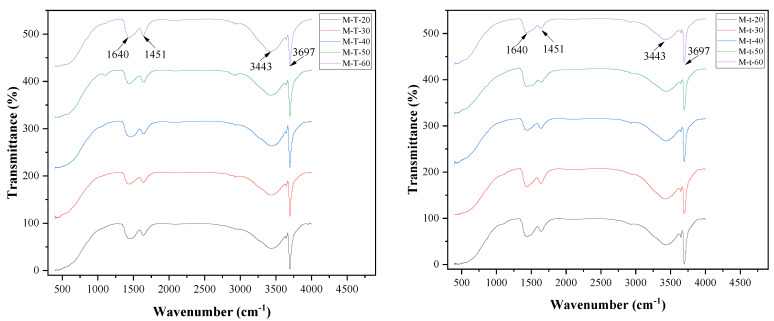
The (**a**) XRD pattern and (**b**) ATR-FTIR spectra of the synthetic nano-Mg(OH)_2_.

As shown in Figure 1a, the XRD patterns indicate that all the diffraction peaks of the samples’ lattice constants are comparable to the values of JCPDS (07-0239) [(001) (100) (101) (102) (110) (111) (103) (202)], and the diffraction peaks are well indexed as the structure of Mg(OH)_2_. In addition, there is no impurity peak except for the characteristic peaks of Mg(OH)_2_, suggesting that the purity of the samples synthesized in this research is fairly high. The data of XRD is shown in Table 3, when the Mg^2+^ concentration is 1.5 mg/mL, and the average size of the grains is the smallest of 23.7 ± 6.7 nm among the samples (M-M-0.5, M-M-1.0, M-M-1.5, M-M-2.0, M-M-3.0). When the reaction temperature and reaction time reached 50 °C and 50 min, the average size of the grains is the smallest of 27.1 ± 9.2 and 24.4 ± 8.4 nm. Thus, most suitable synthesis conditions of the nano-Mg(OH)_2_ powders are Mg^2+^ concentration 1.5 mg/mL, reaction temperature 50 °C and reaction time 50 min, which obtain a smallest crystal grain of the nano-Mg(OH)_2_ (M-M-1.5).

#### 3.1.2. ATR-FTIR Spectra Analysis

The surface groups of the synthetic powders are shown in the ATR-FTIR spectra. It can be seen in Figure 1b that the most obvious sharp and high-intensity absorption peak is located at 3697 cm^−1^, which is the contraction vibration peak of O-H in the crystal structure of Mg(OH)_2_. The characteristic peak of Mg-OH bending vibration is located at 1640 cm^−1^. The characteristic absorption peak representing the bending vibration of -OH is located at 1451 cm^−1^, and another characteristic absorption peak with a broad absorption band is located at 3443 cm^−1^. The reason for the absorption peak is the change of the free proton in Mg(OH)_2_ to the conductive state.

#### 3.1.3. Settling Rate

**Figure 2:**
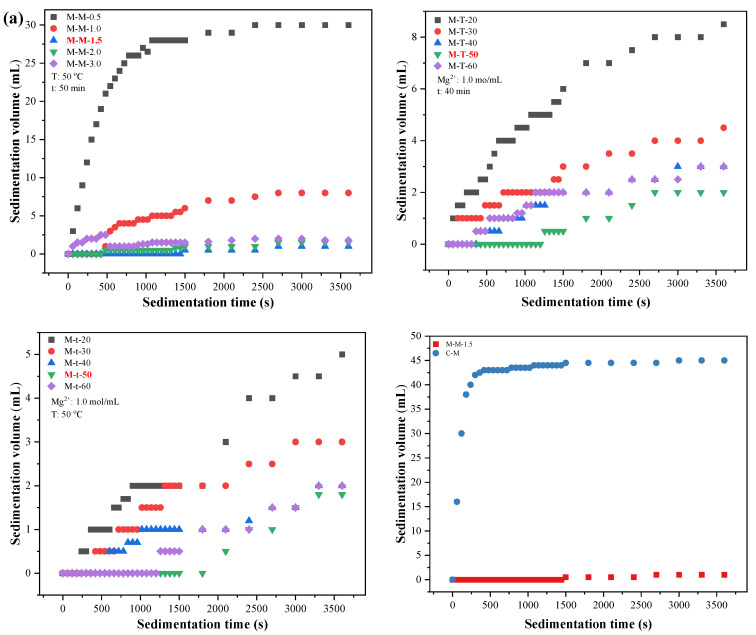

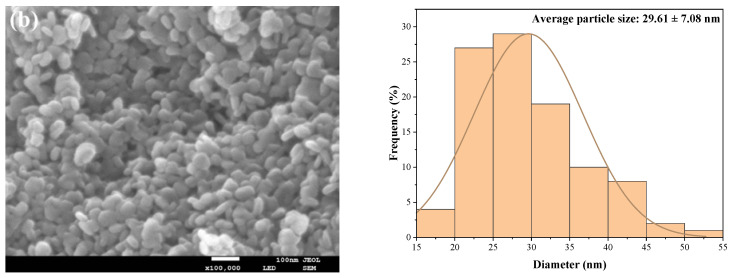
The (**a**) settling rate of synthetic powders and (**b**) SEM image and particle size distribution of M-M-1.5.

Generally, inorganic nanoparticles could agglomerate to a certain point while the degree of aggregation is different. The dispersion of inorganic nanoparticles in water is commonly expressed by the sedimentation volume. The smaller the sedimentation volume is, the slower the settling rate is, which indicates that the dispersion performance of the powder is excellent. On the contrary, the smaller the sedimentation volume, the worse the dispersion performance of the powder. As shown in Figure 2a, the sedimentation volume of M-M-1.5 is slower than that of the samples of M-M-0.5, M-M-1.0, M-M-2.0 and M-M-3.0. The powders at the reaction temperature of 50 °C and the reaction time of 50 min show the more ideal sedimentation volume than that of the others and their reaction temperature and reaction time. In addition, the sedimentation volume of M-M-1.5 is slower than of the C-M. Therefore, the sample of M-M-1.5 exert a minimum sedimentation volume, which shows the best settling rate among all the samples. This is owing to the self-made M-M-1.5 which has an excellent dispersion, while the commercial CM has a poor dispersion so that it is easy to agglomerate.

#### 3.1.4. Microscopic Appearance and Particle Size Distribution

The SEM image and particle size of the M-M-1.5 is shown in Figure 2b. As the M-M-1.5 image shows, the synthetic nano-Mg(OH)_2_ of M-M-1.5 are granular in the form of particles with smooth surfaces, and the dispersion performance of M-M-1.5 is better with less agglomeration. It can be seen from the curve that the particle size distribution of the M-M-1.5 belongs to a normal distribution. The particle size of M-M-1.5 is located within the range of 15–55 nm, and the average particle size is 29.61 ± 7.08 nm. Thus, the nano-Mg(OH)_2_ synthesized by double drop-reverse precipitation method can obtain the advantage of good uniformity and dispersion.

### 3.2. Characterization and Performance of Modified Nano-Mg(OH)_2_

#### 3.2.1. XRD Result

**Figure 3:**
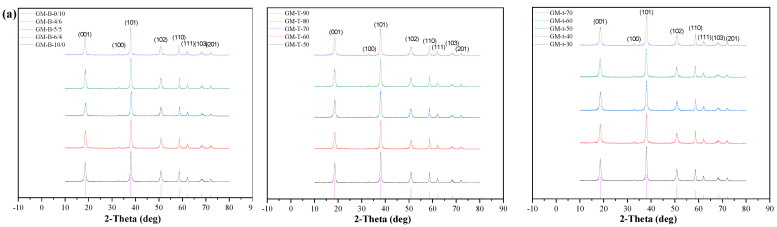

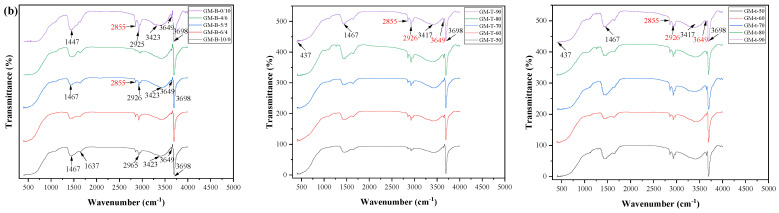
The (**a**) XRD pattern and (**b**) ATR-FTIR spectra of the modified nano-Mg(OH)_2_.

The XRD pattern of the modified nano-Mg(OH)_2_ is shown in Figure 3a, and all the diffraction peaks are well indexed as the structure of Mg(OH)_2_. There is no impure peak other than the characteristic peaks of nano-Mg(OH)_2_ which suggests that the high-purity nano-Mg(OH)_2_ is obtained. Based on the Scherrer equation, the particle sizes of nano-Mg(OH)_2_ corresponding to each diffraction peak are shown in Table 4. The XRD result showed that the grain size of the modified nano-Mg(OH)_2_ becomes larger. When the modifier ratio is 5/5, the temperature is 70 °C and the time is 40 min, the grain size is the smallest of 31.1 ± 5.4 nm.

#### 3.2.2. ATR-FTIR Spectra Analysis

Figure 3b presents the ATR-FTIR spectra of the modified nano-Mg(OH)_2_. The vibrational peaks of nano-Mg(OH)_2_ are located at 3698 cm^−1^ and 437 cm^−1^, corresponding to the stretching vibrations of O-H and Mg-O, respectively. The peaks located at 3423 cm^−1^ and 2952 cm^−1^ are assigned to the -OH and -CH_2_ stretching vibration. The stretching vibration peaks of C-H at 2952 cm^−1^ and 2855 cm^−1^ are enhanced due to the addition of stearic acid modifier. Therefore, the new chemical bonds are formed between the nano-Mg(OH)_2_ and the modifiers.

#### 3.2.3. Activation Index

**Figure 4:**
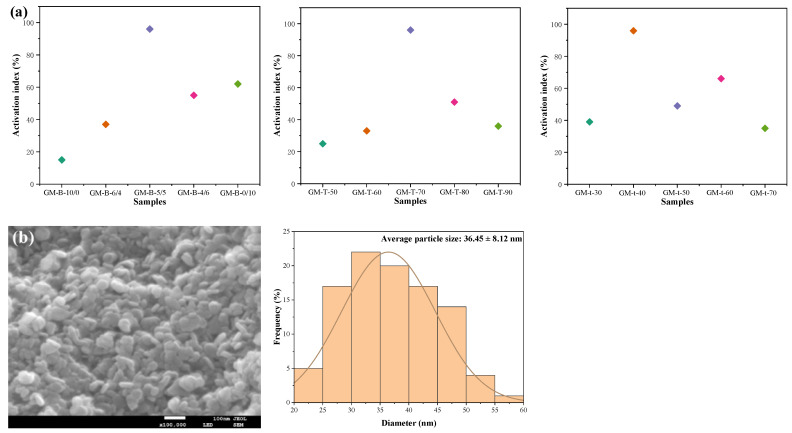
The (**a**) activation index of the modified powders and (**b**) SEM image and particle size distribution of GM-B-5/5.

The activation index of the modified nano-Mg(OH)_2_ is shown in Figure 4, and the higher the activation index, the better the modification effect of nano-Mg(OH)_2_. As shown in the activation index, when the modifier ratio is 5/5, the modification temperature is 70 °C and the modification time is 40 min, the modification effect of the GM-B-5/5 is excellent.

#### 3.2.4. Microscopic Appearance and Particle Size Distribution

The SEM image and particle size of the GM-B-5/5 is shown in Figure 4b. The GM-B-5/5 image indicates that the modified nano-Mg(OH)_2_ is granular in the form of particles with smooth surfaces, and the dispersion performance of GM-B-5/5 is better with less agglomeration. The curve indicates that the particle size distribution of the GM-B-5/5 belongs to a normal distribution. The particle size of GM-B-5/5 is located within the range of 20–60 nm, and the average particle size is 36.45 ± 8.12 nm. Thus, the particle size of the modified powders is larger than that of the powders before modification.

The XRD result indicates that the purity of the synthetic nano-Mg(OH)_2_ and the modified nano-Mg(OH)_2_ in this research are all fairly high, while the grain size of the modified nano-Mg(OH)_2_ (23.7 ± 6.7 nm) becomes a little larger than that of the synthetic nano-Mg(OH)_2_ (31.1 ± 5.4 nm). The ATR-FTIR spectra show that the modified nano-Mg(OH)_2_ stretching vibration peaks of C-H at 2952 cm^–1^ and 2855 cm^–1^ are stronger than that of the synthetic nano-Mg(OH)_2_, which is due to the new chemical bonds which are formed between the nano-Mg(OH)_2_ and the modifier. From the microscopic characterization analysis, it can be seen that the particle size of the modified nano-Mg(OH)_2_ (20–60 nm, 36.45 ± 8.12 nm) is larger than that of the synthetic nano-Mg(OH)_2_ (15–55 nm, 29.61 ± 7.08 nm). Thus, the particle size of the powders is improved during the modification process. Meanwhile, the SEM images showed that the modification process improves the uniformity and the dispersion of the particles.

### 3.3. Characterization and Property of PET Fabrics

#### 3.3.1. ATR-FTIR Spectra Analysis

**Figure 5:**
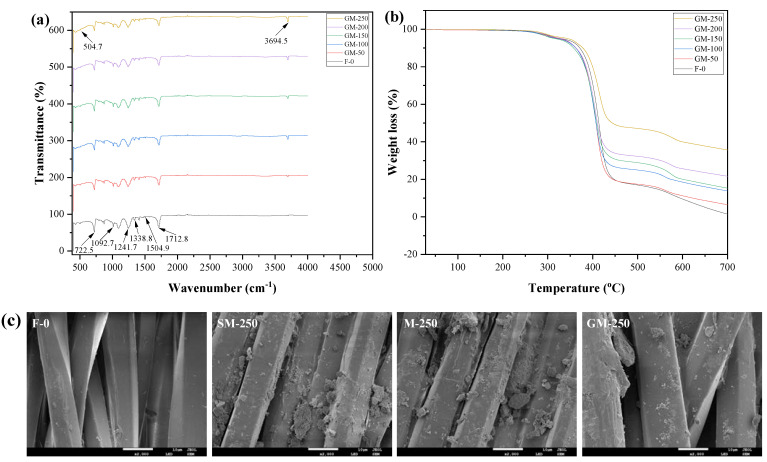
The (**a**) ATR-FTIR spectra, (**b**) TG curves and (**c**) SEM image of the fabrics.

Figure 5a shows the ATR-FTIR spectra of the fabrics. As shown in the spectra, the peaks located at 722.5 cm^–1^ are the bending vibration of the two substituted C=O on benzene ring. The peaks located at 1092.7 cm^–1^ are the vibration of substitution of benzene ring at 1,4–C position. The peaks located at 1241.7 cm^–1^ and 1712.8 cm^–1^ are assigned to the C(O)–O and C=O stretching vibration of the ester group. In addition, the peaks located at 1338.8 cm^–1^–1504.9 cm^–1^ are the vibration of the benzene ring skeleton. Compared to the PET fabrics, the functional PET fabrics have the vibration peaks located at 504.7 cm^–1^ and 3694.5 cm^–1^, which corresponded to the O–H and Mg–O stretching vibration of the modified nano-Mg(OH)_2_.

#### 3.3.2. Thermal Performance

The TGA curves of the fabrics during the decomposition procedure are shown in Figure 5b. The weight loss attributed to water evaporation and thermal decomposition of the fabrics is calculated as a function of temperature. In terms of thermal stability, the fabrics show three stages of weight loss: the F-0 shows the weight loss between 28–340, 340–450 and 450–700 °C, while the functional fabrics show the weight loss between 28–360, 360–455 and 455–700 °C, respectively. At 700 °C, the functional fabrics show the relatively higher residue content than that of the F-0. The residue content of F-0, GM-50, GM-100, GM-150, GM-200 and GM-250 reaches 1.49, 6.45, 13.78, 15.32, 21.68 and 35.69%, respectively. The calculated modified nano-Mg(OH)_2_ content in GM-50, GM-100, GM-150, GM-200 and GM-250 are about 4.97, 7.32, 1.54, 6.36 and 14.01%, respectively, which is probably consistent with the attach ratio of the modified nano-Mg(OH)_2_ in GM-50, GM-100, GM-150, GM-200 and GM-250.

#### 3.3.3. SEM

The SEM images of the fabrics are shown in Figure 5c. The surface of the PET fabric (F-0) is smooth and the fiber diameter is about 9 μm. After loading the CM, there are particulate matters of CM on the fabrics. However, the CM agglomerates seriously on the surface of the fabrics. As shown in the M-250 image, the M agglomerates on the surface of the fabrics, while in the GM-250 image, the GM has a better dispersion on the surface of the fibers. Thus, the modified nano-Mg(OH)_2_ enhance the dispersion performance, which obtain an excellent binding force with the fabrics.

#### 3.3.4. Flame Retardant Property

The flame retardant property of the fabrics is shown in Table 5. The pure PET fabrics have a poor flame retardant property due to the LOI which is 20%, the damaged length is 30 cm, the afterburning time is 38 s and the smoldering time is 0. When loaded with the CM, the flame retardant property of the PET fabrics is improved, and the CM-200 has a good flame retardant property (LOI 23%; damaged length 20 cm; afterburning time 0 s; smoldering time 0 s). When loaded with the M, the flame retardant property of M-200 has a better flame retardant property (LOI 26%; damaged length 13 cm; afterburning time 0 s; smoldering time 0 s). Moreover, when loaded with the GM, the flame retardant property of the GM-150 is excellent (LOI 28%; damaged length 10 cm; afterburning time 0 s; smoldering time 0 s). Therefore, the GM can greatly improve the flame retardant property of the PET fabrics, and the flame retardant effect shows a trend of first increasing and then decreasing with the increase of the GM concentration. The reason may be that when the concentration is too small, it make the flame retardant effect not obvious, while when the concentration is too large, it may cause cracks on the surface of the fabrics, resulting in a poor flame retardant effect of the fabrics. Thus, a suitable concentration of the GM is 150 g/L. The GM on the fabrics can be decomposed into MgO and water vapor when heated. On the one hand, the MgO will be deposited on the surface and inside of the fibers, forming an inorganic protective film that blocks combustible gases and heat, thereby preventing combustion. On the other hand, the water vapor will reduce the concentration of combustible gases and prevent combustion.

#### 3.3.5. Antibacterial Property

**Figure 6:**
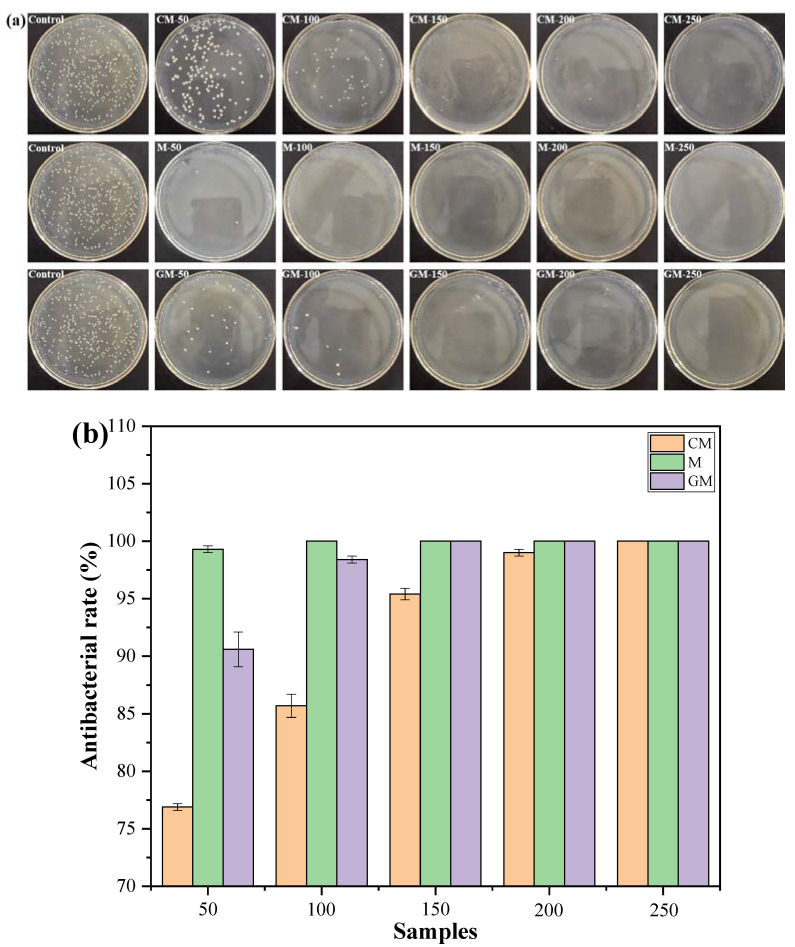
The (**a**) *E. coli* colony on agar plates and (**b**) antibacterial rate of the fabrics.

Figure 6a,b and Table 6 show the antibacterial property against *E. coli* of the fabrics. The *E. coli* colony can be visually observed on the LB agar plates of control, which is covered with *E. coli* colony (307 ± 5.9), while the *E. coli* colony number decreases as the content of the powders on the PET fabrics. When the content of the CM reaches 250 g/L, there is no colony on the LB agar plates (CM-250). As shown in Figure 6b, the reduction percentage of the *E. coli* colony number is calculated, and the antibacterial rate of CM-50, CM-100, CM-150, CM-200 and CM-250 reached 76.9 ± 0.3, 85.7±1.0, 95.4 ± 0.5, 99 ± 0.3 and 100 ± 0%, respectively. When the content of the M reaches 100 g/L, the CM-100 has no colony on the LB agar plates, and the antibacterial rate is 100%. When the content of the GM reaches 150 g/L, the GM-150 has no colony on the LB agar plates, and the antibacterial rate is 100%. The above results indicate that the fabrics have a certain antibacterial property against *E. coli*, which entirely depended on the powder content on the fabrics. Moreover, the antibacterial property are as follows: M > GM > CM. Thus, the modification process slightly reduced the antibacterial property of the synthetic nano-Mg(OH)_2_.

#### 3.3.6. Breaking Strength

The breaking strength of the fabrics are listed in Table 7 with the F-0 as a control. The breaking strength is slightly increased with the increase of powder content on the PET fabrics, which indicates that the addition of the powders can enhance the breaking strength of the PET fabrics. The results demonstrate that modification of the PET fabrics using nano-Mg(OH)_2_ has almost no obvious effect on the breaking strength of the PET fabrics, and the functional PET fabrics have an excellent physico-mechanical property as the pure PET fabrics.

## 4. Conclusions

In summary, we have demonstrated a double drop-reverse precipitation strategy to synthesize the nano-Mg(OH)_2_ with small particle size and high dispersibility. When the synthesis conditions are: Mg^2+^ concentration 1.5 mg/mL, reaction temperature 50 °C and reaction time 50 min, the nano-Mg(OH)_2_ result in a crystal grain of 23.7 ± 6.7 nm. The synthetic nano-Mg(OH)_2_ was modified by titanate coupling agents and stearic acid in order to obtain the powders which are compatible to the hydrophobic PET fabrics. When the modifier ratio is 5/5, modification temperature is 70 °C and modification time is 40 min, the modification effect is excellent. After loading the modified nano-Mg(OH)_2_ on PET fabrics through dip-coating technology, the flame retardant property of the GM-150 included LOI 28%; damaged length 10 cm; afterburning time 0 s; smoldering time 0 s. Additionally, the antibacterial rate of the GM-150 against *E. coli* reached 100%. Therefore, this work has developed a simple method to fabricate multifunctional PET fabrics with excellent flame retardant and antibacterial properties.

## Figures and Tables

**Table 1:** The sample labels and reaction conditions of the synthetic nano-Mg(OH)_2_.

Samples	Mg^2+^ (mol/L)	T (°C)	*t* (min)
M-M-0.5	0.5	50	50
M-M-1.0	1.0	50	50
M-M-1.5	1.5	50	50
M-M-2.0	2.0	50	50
M-M-3.0	3.0	50	50
M-T-20	1.0	20	40
M-T-30	1.0	30	40
M-T-40	1.0	40	40
M-T-50	1.0	50	40
M-T-60	1.0	60	40
M-t-20	1.0	30	20
M-t-30	1.0	30	30
M-t-40	1.0	30	40
M-t-50	1.0	30	50
M-t-60	1.0	30	60

**Table 2:** The sample labels and reaction conditions of the nano-Mg(OH)_2_ powders.

Samples	Mass Ratio	T (°C)	*t* (min)
GM-B-10/0	10/0	70	40
GM-B-6/4	6/4	70	40
GM-B-5/5	5/5	70	40
GM-B-4/6	4/6	70	40
GM-B-0/10	0/10	70	40
GM-T-50	5/5	50	40
GM-T-60	5/5	60	40
GM-T-70	5/5	70	40
GM-T-80	5/5	80	40
GM-T-90	5/5	90	40
GM-t-30	5/5	70	30
GM-t-40	5/5	70	40
GM-t-50	5/5	70	50
GM-t-60	5/5	70	60
GM-t-70	5/5	70	70

**Table 3:** The XRD parameters and grain size of the synthetic nano-Mg(OH)_2_.

Samples	Size (nm)								Average Size (nm)
	001	100	011	012	110	111	103	201	
M-M-0.5	20.956	37.262	23.369	21.082	28.342	26.655	37.424	28.568	28.0 ± 6.1
M-M-1.0	26.000	43.364	28.352	22.915	38.129	32.567	24.470	38.144	31.7 ± 7.0
M-M-1.5	17.307	18.286	37.421	27.750	17.183	29.044	20.917	21.381	23.7 ± 6.7
M-M-2.0	15.361	35.160	19.278	16.376	37.227	32.544	29.331	30.264	26.9 ± 8.1
M-M-3.0	20.452	16.535	34.284	34.773	34.059	33.273	18.524	43.050	29.4 ± 8.9
M-T-20	18.522	55.252	25.201	21.519	36.117	29.928	24.726	34.999	30.8 ± 10.9
M-T-30	18.772	49.688	24.073	22.474	44.110	31.666	20.944	39.846	31.4 ± 11.0
M-T-40	26.000	43.364	28.352	22.915	38.129	32.567	24.470	38.144	31.7 ± 7.0
M-T-50	19.239	41.965	21.099	17.733	40.274	30.813	17.910	27.806	27.1 ± 9.2
M-T-60	16.062	54.406	20.056	14.823	39.048	39.048	27.944	21.163	29.1 ± 13.0
M-t-20	17.778	74.138	24.570	21.343	39.538	30.383	22.636	37.243	33.5 ± 17.0
M-t-30	15.293	22.524	21.524	40.980	33.327	18.055	34.973	35.251	27.7 ± 8.9
M-t-40	18.772	49.688	24.073	44.110	31.666	20.944	39.846	22.474	31.4 ± 11.0
M-t-50	16.182	35.686	21.038	20.338	15.107	40.349	24.371	22.278	24.4 ± 8.4
M-t-60	67.177	19.284	16.754	37.069	30.674	27.172	14.916	68.577	35.2 ± 20.1

**Table 4:** The XRD parameters and grain size of the modified nano-Mg(OH)_2_.

Samples	Size (nm)								Average Size (nm)
	001	100	011	012	110	111	103	201	
GM-B-10/0	26.146	54.205	30.820	28.031	41.115	38.241	30.965	40.608	36.3 ± 8.6
GM-B-6/4	23.982	58.171	30.432	25.620	43.143	34.985	24.149	41.706	35.3 ± 11.2
GM-B-5/5	21.588	52.221	26.791	24.669	38.900	27.693	26.181	31.803	31.2 ± 9.3
GM-B-4/6	26.295	52.227	30.625	28.292	43.400	37.349	27.937	43.748	36.2 ± 8.9
GM-B-0/10	26.295	52.611	32.115	26.059	41.963	39.148	32.420	38.903	36.2 ± 8.3
M-T-50	26.146	54.205	30.820	28.031	41.115	38.241	30.965	40.608	36.3 ± 8.6
M-T-60	22.508	49.861	28.688	23.373	40.246	35.820	23.432	33.064	32.1 ± 9.0
M-T-70	22.766	35.686	29.746	24.865	40.453	34.213	28.662	32.745	31.1 ± 5.4
M-T-80	23.339	36.506	29.992	23.850	41.087	34.286	24.987	40.194	31.8 ± 6.8
M-T-90	22.435	63.885	28.745	24.704	40.453	35.582	28.662	33.721	34.8 ± 12.3
M-t-30	26.146	54.205	30.820	28.031	41.115	38.241	30.965	40.608	36.3 ± 8.6
M-t-40	21.417	48.108	27.493	24.784	38.579	34.733	22.882	32.494	31.3 ± 8.5
M-t-50	23.339	56.562	28.802	23.888	40.873	31.706	29.115	34.758	33.6 ± 10.2
M-t-60	22.991	54.205	27.597	26.654	41.741	40.029	26.171	31.122	33.8 ± 9.9
M-t-70	22.079	59.626	28.631	24.906	42.416	31.457	24.837	39.910	34.2 ± 11.8

**Table 5:** The flame retardant property of the fabrics.

Samples	LOI (%)	Damaged Length (cm)	Afterburning Time (s)	Smoldering Time (s)
F-0	20	30	38	0
CM-50	20	30	22.7	0
CM-100	20	30	18	0
CM-150	21	25	0	0
CM-200	23	20	0	0
CM-250	20	30	0	0
M-50	20	30	29.3	0
M-100	21	30	30.6	0
M-150	23	20	1.3	0
M-200	26	13	0	0
M-250	20	30	0	0
GM-50	20	30	0	0
GM-100	21	30	0	0
GM-150	28	10	0	0
GM-200	24	15	0	0
GM-250	21	30	0	0

**Table 6:** The *E. coli* colony numbers of the fabrics on the agar plates.

Samples	Control	CM-50	CM-100	CM-150	CM-200	CM-250
Colonies number	307 ± 5.9	71 ± 0.8	44 ± 2.9	14 ± 1.6	3 ± 0.8	0 ± 0
Samples	Control	M-50	M-100	M-150	M-200	M-250
Colonies number	307 ± 5.9	2 ± 0.8	0 ± 0	0 ± 0	0 ± 0	0 ± 0
Samples	Control	GM-50	GM-100	GM-150	GM-200	GM-250
Colonies number	307 ± 5.9	29 ± 4.5	5 ± 0.8	0 ± 0	0 ± 0	0 ± 0

**Table 7:** The flame retardant property of the fabrics.

Samples	Breaking Strength (N)		Elongation at Break (%)	
	Vertical	Weft	Vertical	Weft
F-0	465.1	341.3	31.4	16.9
CM-50	460.3	330.5	30.3	15.7
CM-100	466.7	340.2	30.2	16.8
CM-150	468.2	343.2	31.8	17.8
CM-200	473.2	343.8	31.6	16.2
CM-250	473.3	346.0	30.6	17.3
M-50	476.8	340.3	32.5	17.4
M-100	463.5	345.2	30.6	18.6
M-150	467.6	350.8	30.6	18.4
M-200	467.6	350.2	31.8	19.6
M-250	470.8	348.3	32.4	19.5
GM-50	466.2	343.3	31.3	16.3
GM-100	466.8	345.2	31.7	18.4
GM-150	470.3	342.3	32.0	19.6
GM-200	470.8	347.8	32.7	19.5
GM-250	473.2	350.2	33.3	17.2
